# Total Hip Arthroplasty in Hip Osteoarthritis with Subtrochanteric Localized Periosteal Thickening: Preoperative Planning Using Finite Element Analysis to Determine the Optimal Stem Length

**DOI:** 10.3390/jcm13195872

**Published:** 2024-10-01

**Authors:** Koshiro Shimasaki, Tomofumi Nishino, Tomohiro Yoshizawa, Ryunosuke Watanabe, Fumi Hirose, Shota Yasunaga, Hajime Mishima

**Affiliations:** Department of Orthopaedic Surgery, Institute of Medicine, University of Tsukuba, 1-1-1 Tennodai, Tsukuba 305-8575, Ibaraki, Japan; koshiro19881020@tsukuba-seikei.jp (K.S.); tyoshizawa@tsukuba-seikei.jp (T.Y.); ryuwatanabe@tsukuba-seikei.jp (R.W.); f.ochiai.0023@tsukuba-seikei.jp (F.H.); syasunaga@tsukuba-seikei.jp (S.Y.); hmishima@tsukuba-seikei.jp (H.M.)

**Keywords:** atypical femoral fracture, finite element analysis, localized periosteal thickening, osteoarthritis, total hip arthroplasty

## Abstract

**Background:** Owing to the risk of atypical femoral fractures, total hip arthroplasty presents unique challenges for patients with ipsilateral osteoarthritis and localized periosteal thickening in the femoral subtrochanteric region. Stem length selection is critical for minimizing stress concentration in the thickened cortex to avoid such fractures. Herein, we report the case of a 78-year-old woman with ipsilateral hip osteoarthritis and localized subtrochanteric periosteal thickening. **Methods:** Preoperative planning included a finite element analysis to assess the stress distribution across various stem lengths. A simulation was conducted to determine the optimal stem length to span the cortical thickening and reduce the risk of postoperative complications. **Results**: The finite element analysis indicated that a stem length of >150 mm was required to effectively reduce the stress at the site of cortical thickening. A 175 mm stem was selected for total hip arthroplasty, which provided a favorable stress distribution and avoided the risk of stress concentration. **Conclusions:** In cases of ipsilateral osteoarthritis with localized subtrochanteric periosteal thickening, finite element analysis can be valuable for preoperative planning to determine the optimal stem length, thereby reducing the risk of atypical femoral fractures. Further studies with multiple cases are recommended to validate these findings and improve surgical outcomes.

## 1. Introduction

The 2013 revision of the American Society for Bone and Mineral Research Task Force defines the localized periosteal thickening of the femoral lateral cortex as a primary diagnostic criterion for atypical femoral fractures (AFFs) [1]. To avoid stress concentration, surgical interventions for an AFF generally involve using the longest possible intramedullary nails. Localized periosteal thickening can progress to AFFs even with minor trauma or low-energy impacts. For localized periosteal thickening, conservative treatment focuses on weight restriction, and preventive intramedullary nailing has been reported to achieve high bone-healing rates of 95–98% [2]. However, once the progression to an AFF occurs, treatment can be challenging. In cases of AFFs, femoral bowing or a narrow medullary canal could be present. The intramedullary nails used tend to be thinner and shorter, which often raises concerns of having an insufficient fixation strength at the fracture site. Additionally, mismatches between femoral geometry and the implant can lead to poor lower limb alignment and gaps at the fracture site, potentially resulting in delayed union or non-union [3]. Reports indicate that 26–39% of AFFs treated surgically experience delayed union or non-union, with the risk of re-operation being approximately three times higher compared to standard femoral fractures [1,4]. Therefore, careful and appropriate management to prevent progression from localized periosteal thickening to an AFF is crucial.

Min et al. proposed a scoring system to assess the risk of progression to AFFs based on four factors: (1) location, (2) pain, (3) contralateral fracture presence and severity, and (4) radiolucent lines, suggesting criteria for preventive intramedullary nailing [2]. Similarly, Joaquin et al. classified localized periosteal thickening using computed tomography (CT) and magnetic resonance imaging into categories such as ‘stress reaction’, ‘stress fracture’, ‘incomplete AFF’, and ‘complete AFF’, and proposed treatment strategies for each category [5]. However, no comprehensive consensus on the appropriate surgical indications or timing for intervention exists.

In cases of localized periosteal thickening or an AFF combined with ipsilateral osteoarthritis, surgery should be performed using a stem rather than an intramedullary nail. Total hip arthroplasty (THA) in the presence of ipsilateral osteoarthritis with localized cortical thickening is rare, with few reported cases. In a similar case report to that of ours, a stem of insufficient length was used, leading to the development of an AFF postoperatively [5]. Although the stem tip might be positioned near the area of cortical thickening, potentially increasing the risk of an AFF due to concentrated stress, no studies have specifically addressed stem selection from the perspective of stress distribution.

Herein, we describe our experience in performing THA in a patient with ipsilateral osteoarthritis of the hip and localized cortical thickening in the subtrochanteric region of the femur. The procedure planning was guided by a preoperative finite element method (FEM) simulation to assess the femoral stress distribution.

In this study, we focused on stem length and conducted three-dimensional (3D) simulations using FEM to evaluate stress distributions in stems of varying lengths, ultimately guiding our stem selection. Moreover, we considered and emphasized treatment strategies for ipsilateral osteoarthritis with localized cortical thickening. We hypothesized that the longer the bridging length of the stem over the thickened cortex, the greater the stress reduction in that area. To the best of our knowledge, this study is the first to examine the relationship between stem length and stress distribution in local femoral cortical thickening using the FEM.

## 2. Case Presentation

A 78-year-old woman without a history of developmental hip dysplasia had been diagnosed with systemic lupus erythematosus at 46 years of age. The initial treatment involved steroid pulse therapy, followed by continuous oral prednisolone administration thereafter. Concurrently, treatment with 200 mg etidronate was initiated for glucocorticoid-induced osteoporosis. After 47 days, the medication was switched from etidronate to 35 mg alendronate, which was continued thereafter.

**Figure 1:**
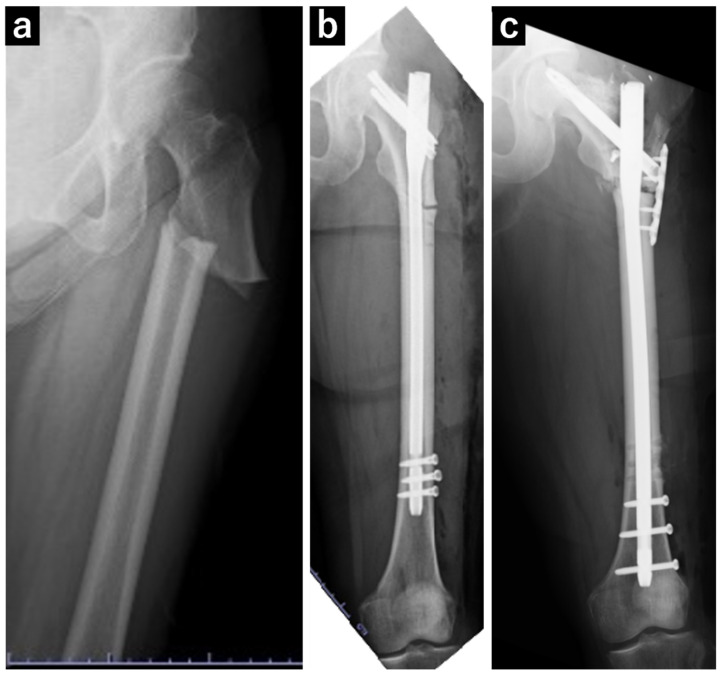
Plain radiograph of the left femur. (**a**) A transversal fracture in the subtrochanteric region of the left femur diagnosed as an atypical femoral fracture. (**b**) Postoperative image following open reduction and internal fixation of the atypical femoral fracture. (**c**) Postoperative image following surgery for pseudarthrosis of the left femur, in which replacement with a long nail and additional plate fixation were performed.

**Figure 2:**
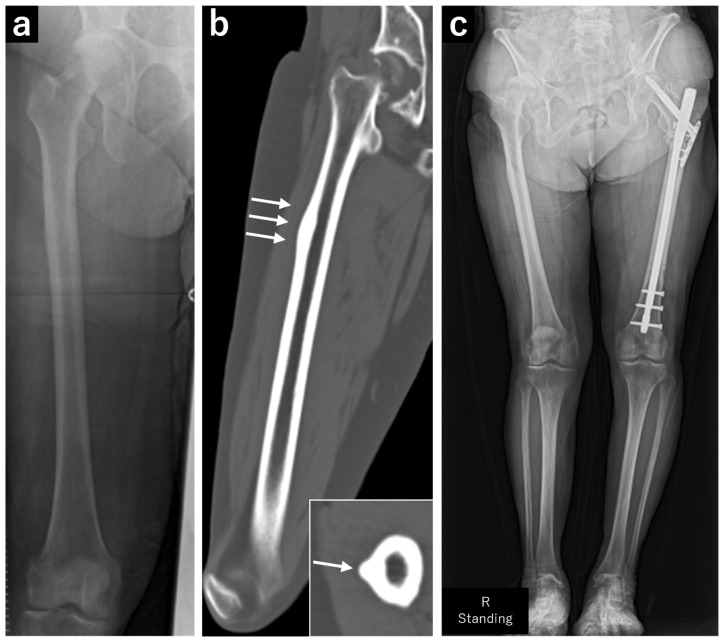
Plain radiograph and computed tomography (CT) images of the right femur. (**a**) Plain radiograph showing localized cortical hypertrophy at the subtrochanteric region. (**b**) Plain CT image showing no obvious radiolucent lines. The white arrows indicate localized periosteal thickening in the coronal and axial planes, respectively. (**c**) A full-length plain radiograph of the lower limbs showing no significant femoral bowing deformity.

At 73 years of age, the patient experienced a fall and was diagnosed with a left subtrochanteric AFF based on plain radiography and CT findings. Additionally, localized lateral cortical thickening was observed in the ipsilateral subtrochanteric region, along with osteoarthritis of the right hip (Figure 1). The patient underwent open reduction and internal fixation with an intramedullary nail for the left subtrochanteric AFF. Alendronate was discontinued, and treatment comprising 600 µg teriparatide (recombinant) was initiated, which was continued for 2 years. Due to non-union at the fracture site, the patient underwent surgery for pseudarthrosis at 75 years of age (Figure 2). Given the gradual worsening of the right hip osteoarthritis, THA was planned when the patient turned 78 years of age.

Informed consent was obtained from the patient for using and publishing these data, and this retrospective study was approved by the ethics review committee of our institution (approval code: H27-041).

## 3. Materials and Methods

### 3.1. Finite Element Analysis

We performed preoperative 3D simulations using FEM to analyze stress distributions in the femur following stem insertion. We also evaluated the stress distributions at the site of cortical thickening for different stem lengths.

### 3.2. Three-Dimensional Modeling of the Femur and Implant

Preoperative CT was performed using a 256-slice multidetector CT scanner (Brilliance iCT; Philips Healthcare, Cleveland, OH, USA). The scan, which included a bone mineral density phantom (QRM-BDC/3 Phantom, QRM Quality Assurance in Radiology and Medicine GmbH, Möhrendorf, Germany), captured images from the pelvis to both knee joints (120 kV/166 mAs, 1 mm thin slice).

First, based on CT images, we simulated stem insertion using the ZedHip 3D preoperative planning software (version 17.0, Lexi Co., Ltd., Tokyo, Japan). The patient in this case had a narrow medullary canal and mild anterior bowing of the femoral shaft. As the selection of stems that could span the area of cortical hypertrophy and fit the femoral morphology of the patient was limited, we opted for the Arcos^®^ One-piece Femoral Revision System (Zimmer, Warsaw, IN, USA) with a 9.5 mm diameter and 175 mm length, in the high offset configuration.

**Figure 3:**
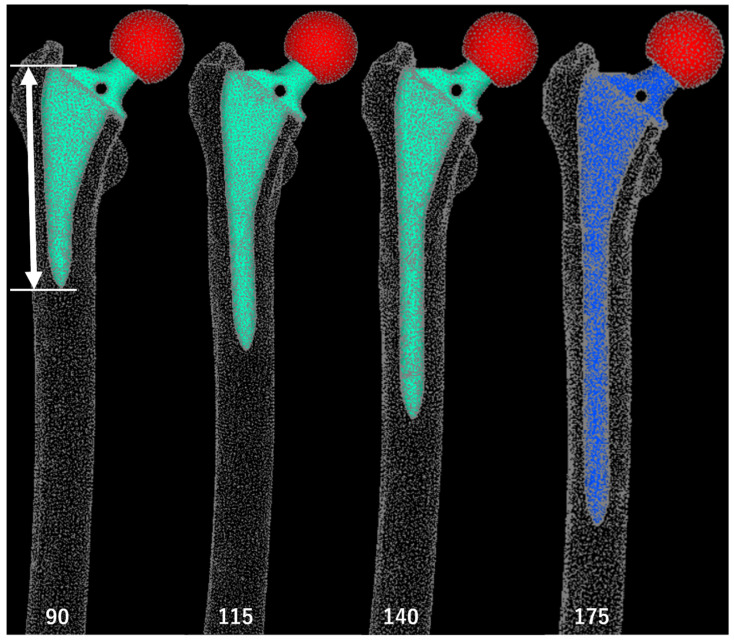
Standard Triangulated Language data for stems of varying lengths. White arrows: stem lengths. The 90 mm stem length corresponds to a position 25 mm proximal to the thickened region (equivalent to 1 femoral cortical diameter), the 115 mm stem length corresponds to the level of the thickened cortical region, and the 140 mm stem length corresponds to 25 mm (1 femoral cortical diameter) bridging length over the thickened area. A 175 mm stem was used in the actual surgery.

We then generated Standard Triangulated Language (STL) data for stems of varying lengths. The original stem STL data were imported into the 3D computer-aided design software Fusion 360 (v.2.0.20256, Autodesk, San Francisco, CA, USA) and modified using the “cut” and “join” functions. The stem lengths included the original size of 175 mm, a 115 mm stem aligned with the level of cortical thickening at the stem tip, and additional lengths ranging from 90 mm to 180 mm in 10 mm increments (Figure 3). The 90 mm stem was the shortest length that could be generated without altering the proximal stem geometry, while 180 mm was the maximum length that could be inserted with the same alignment.

Next, we constructed a 3D model of the right femur. Preoperative CT data were imported into the FEM analysis software MECHANICAL FINDER (MF, version 13.0, Extended Edition, Research Center of Computational Mechanics, Tokyo, Japan), in which bone was defined as an area with a density of ≥300 Hounsfield units (HUs). Only the right femur was extracted. We generated a model of the femur by performing an osteotomy at the proximal level 10 mm above the lesser trochanter and creating an isosurface mesh to represent the external shape of the femur.

**Figure 4:**
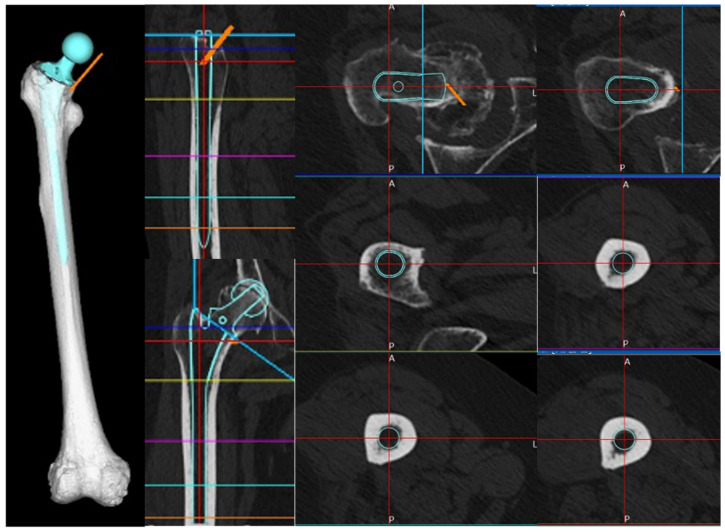
Stem insertion depth and alignment. Preoperative planning was performed using three-dimensional (3D) ZedHip software. The stem was planned to be inserted at a 32° anteversion angle relative to the condylar axis on the femoral horizontal section, a 3° valgus angle relative to the femoral axis on the femoral coronal section, a 5° flexion angle relative to the femoral axis on the femoral sagittal section, and a depth of 11 mm from the apex of the greater trochanter.

By registering the acquired stem STL data with a 3D model of the right femur in MF, we were able to replicate femur post-stem insertion. The insertion depth and alignment of the stem were reproduced in MF based on the simulation conducted in ZedHip, ensuring consistency in 3D coordinates across the analysis of each stem (Figure 4).

### 3.3. Material Properties

We used four-node tetrahedron elements for the solid components. For stress analysis, shell elements with a thickness of 0.001 mm were applied to the bone surface to ensure that they did not affect bone strength. We conducted mesh convergence tests to determine the mesh size.

### 3.4. Material Parameters

For 3D femoral model, we set the internal mesh size to minimum and maximum values of 1 mm and 2 mm, respectively, and employed a heterogeneous material model for the bone properties. The Young’s modulus was derived using bone mineral density (BMD, ρ (g/cm^3^)), calculated from the CT values (HU) based on an assumed linear relationship [6,7]. Then, the values were estimated using Keyak’s predictive transformation formula and incorporated into the model [8]. For artificial components, we set the internal mesh size to a minimum of 0.5 mm and a maximum of 1 mm. This study used a model with homogeneous material. The stem was modeled using the material properties of a titanium alloy (Ti-6Al-4V). For the artificial head, we used ceramic (alumina) [6] (Table 1).

### 3.5. Loading and Boundary Conditions

**Figure 5:**
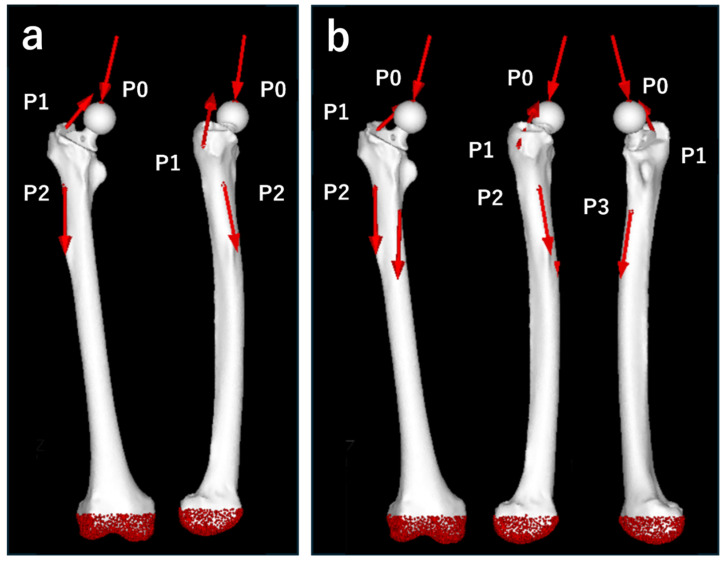
Loading points and fixation sites for simulated normal walking and stair climbing. (**a**) Normal walking. P0: hip contact point; P1: the combined force of the abductor muscles and iliotibial band; and P2: action point of the vastus lateralis. (**b**) Stair climbing. P0: hip contact point; P1: combined force of the abductor muscles, iliotibial band, and tensor fasciae latae; P2: action point of the vastus lateralis; and P3: action point of the vastus medialis. In both conditions, the distal region of the femur, shown in red, was fully constrained.

We designed the loading conditions to simulate the forces encountered during daily activities. We applied the maximum loads corresponding to “normal walking” and “stair climbing” based on previous reports [9,10]. The “stair-climbing” scenario allowed for the examination of higher load forces and stronger torques than “normal walking”. The magnitude of each vector was determined by the patient’s body weight (50.4 kg), with the load acting as shown in Table 2 and Figure 5.

For the constraint conditions, the distal part of the femur was fixed (Figure 4). The boundary conditions included a fixed connection between the stem and head and a contact condition with a friction coefficient of 0.49 between the femur and stem [11].

### 3.6. Static Structural Analysis

**Figure 6:**
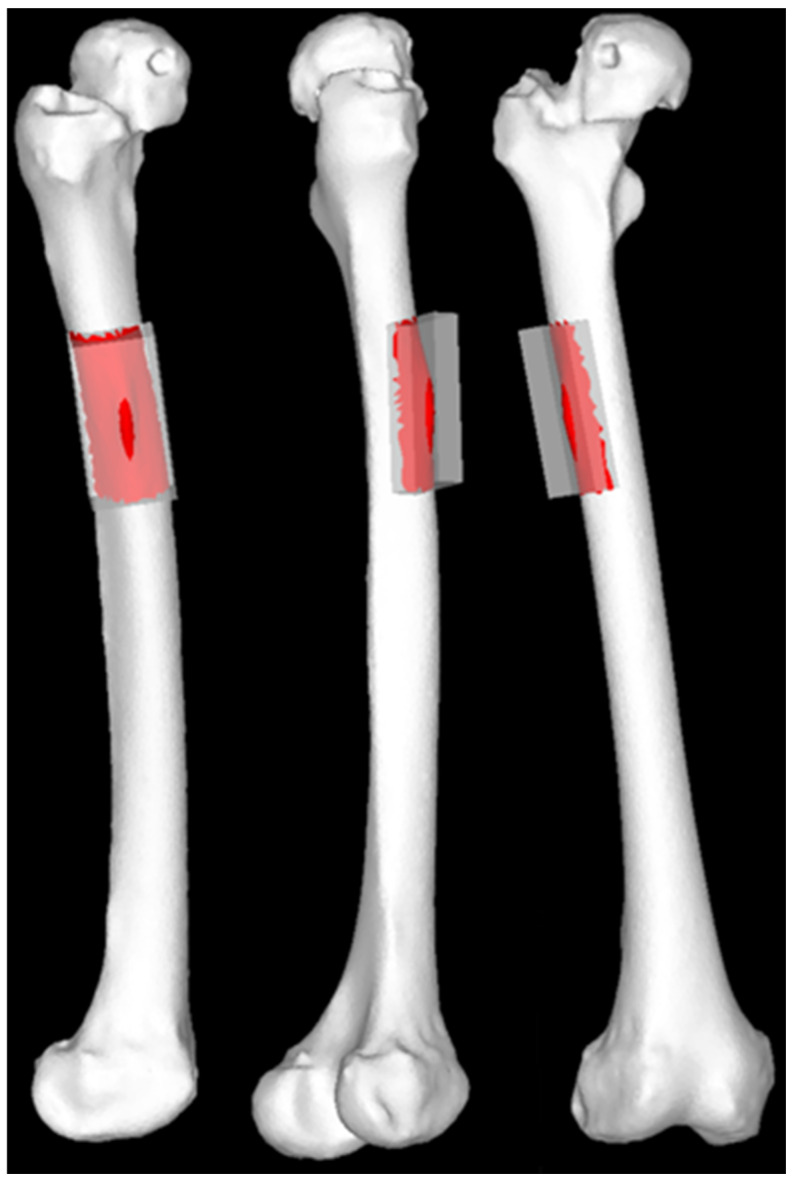
Regions of interest for stress value calculations. The regions of interest included the femoral surface shell elements within a 40 mm wide and 60 mm long axial area centered around the apex of the area of cortical hypertrophy to encompass the entire hypertrophic area.

We conducted simulations under normal walking and stair-climbing conditions using stems of varying lengths. Elastic analysis was performed with the load gradually increasing linearly, and linear static analysis was applied to the calculations. We used equivalent stress (MPa) as the measure of stress and calculated the overall stress distributions across the femur, as well as the average and maximum equivalent stress values in the area of cortical thickening. The hypertrophic region was standardized to 40 mm in width and 60 mm in length along the bone axis, centered around the apex of the hypertrophic area to encompass the entire hypertrophic region. The calculations considered only the stress values of the femoral surface shell elements (Figure 6).

## 4. Results

The number of elements comprising each model is shown in Table 3.

**Figure 7:**
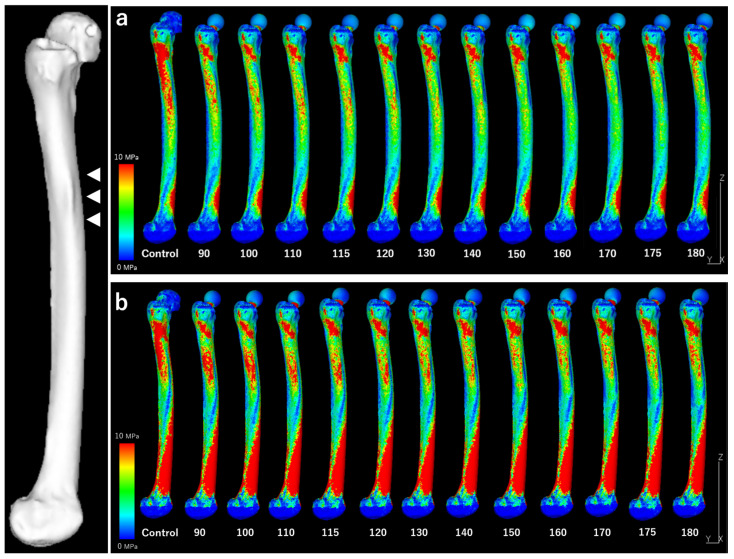
Stress distributions across the entire lateral femoral surface. White arrow: Localized periosteal thickening. (**a**) “Normal walking”. (**b**) “Stair climbing”. Under both conditions, the stress around the cortical hypertrophy area decreased as the stem length increased. No new stress concentrations are observed.

**Figure 8:**
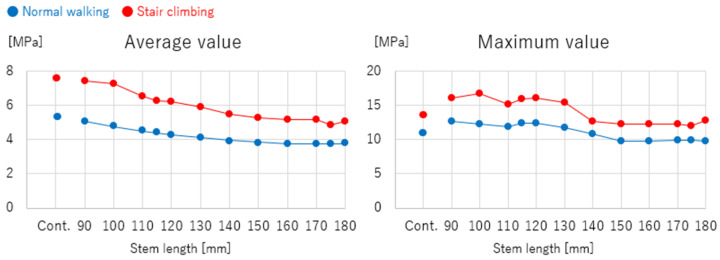
Stem lengths and equivalent stresses in the cortical hypertrophic areas. The equivalent stresses in the cortical hypertrophic areas followed trends closely approximated by curves as a function of stem length.

Figure 7 shows the stress distributions across the lateral surface of the femur for each model during normal walking and stair-climbing conditions. Figure 8 illustrates the mean and maximum equivalent stress values in the region of cortical thickening. The stress distribution across the lateral surface of the femur decreased following stem insertion. No new areas of stress concentration were observed during normal walking or stair-climbing conditions.

The mean equivalent stress at the thickened region decreased gradually as the stem length increased, following an approximation curve. The stress nearly plateaued at a length of 120 mm during normal walking and 150 mm during stair climbing, with approximately the same level as the control. Similarly, the maximum stress decreased with increasing stem length, following a trend similar to the mean values. However, elevated stress levels were observed near a stem length of 115 mm, where the stem tip overlapped with the thickened region during normal walking and stair-climbing conditions. Beyond this length, where the stem tip extended past the thickened area, the stress followed a decreasing approximation curve. The stress values plateaued at a length of approximately 150 mm, which was almost identical to that of the control.

**Figure 9:**
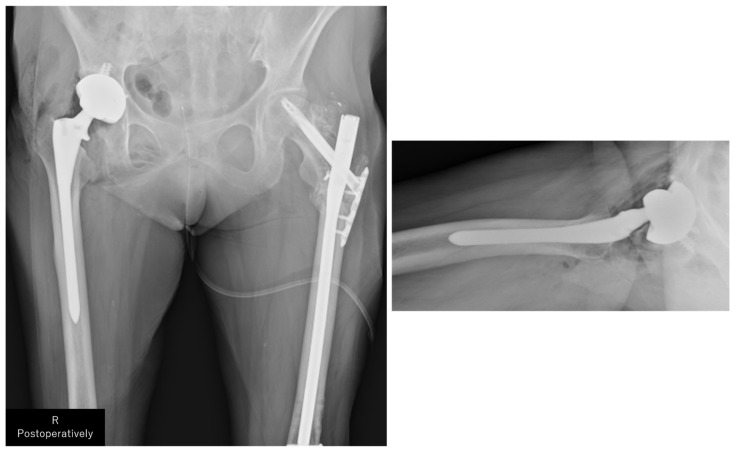
Plain radiograph of the hip joint immediately postoperatively. The stem (Arcos^®^ One-piece Femoral Revision System, Zimmer, Warsaw, IN, USA, φ9.5 mm × 175 mm, high offset) was inserted to a depth of 11 mm below the apex of the greater trochanter, as planned preoperatively. The cup (Continuum^®^, Zimmer, Warsaw, IN, USA, Φ28/46 mm) was fixed and positioned using four screws. The surgery was completed in 1 h 20 min, with a blood loss of 50 mL.

Based on the abovementioned findings, we performed THA using a posterior approach (Figure 9). Postoperatively, full weight bearing on the affected limb was allowed. The patient could walk with a cane and was transferred to another hospital for rehabilitation on postoperative day 14. At the final follow-up (3 months postoperatively), the patient could walk independently and experienced no difficulties in her daily activities.

## 5. Discussion

The preoperative 3D simulation conducted using the FEM to evaluate the stress distribution at the site of localized cortical thickening revealed reduced stress along the femur’s lateral surface after the stem insertion, without introducing new stress concentrations during normal walking or stair-climbing conditions. The stress at the cortical thickening progressively decreased and stabilized at approximately 120 mm for walking and 150 mm for stair climbing. The maximum stress initially peaked near the 115 mm stem length, where the stem tip corresponded with the cortical thickening but diminished and plateaued with longer stems, particularly beyond 150 mm.

AFFs tend to develop gaps on the medial side of the fracture because of the mismatch between femoral morphology and implants, making non-union more likely [4,12,13]. Furthermore, the long-term use of bone resorption inhibitors; the over-suppression of bone turnover due to metabolic diseases; and decreased bone quality associated with collagen diseases, rheumatoid disorders, or glucocorticoid use can all contribute to delayed bone healing [12,14,15]. The subtrochanteric region is particularly challenging because it consists predominantly of cortical bone with a poor blood supply, making the stable fixation of the fracture challenging. Additionally, adequate medial support is difficult to secure due to the transverse or oblique nature of AFFs. These anatomical complexities make bone healing difficult, rendering subtrochanteric AFFs particularly refractory [3]. Therefore, the strict management of femoral cortical hypertrophy to prevent progression to an AFF is crucial not only for maintaining patients’ activities of daily living and quality of life, but also from an economic healthcare perspective.

Conservative treatment for femoral cortical hypertrophy primarily involves discontinuing bisphosphonate (BP) therapy and imposing weight-bearing restrictions on the affected limb [12]. The risk of AFF progression decreases rapidly following BP therapy cessation [16]. Additionally, PTH therapy may effectively promote bone healing in cases of AFFs [17]. Although surgical treatment with intramedullary nailing for femoral cortical hypertrophy has a high bone union rate of 95–98% [2], a clear consensus is lacking regarding the indications for prophylactic surgery or the optimal timing for such interventions.

In the present case of localized cortical hypertrophy, the Min BW scoring system resulted in a total of six points (two, two, one, and one for location, pain, contralateral fractures, and radiolucent lines, respectively), which is below the threshold of eight points for recommending prophylactic surgery. According to the flowchart proposed by Joaquin et al., the preoperative plain CT scan did not reveal lucent lines, suggesting that the condition could be classified as either a “stress reaction” or “stress fracture”.

Based on this assessment, we concluded that a surgical intervention was not warranted for localized cortical hypertrophy. However, given the risk of stress concentration in the hypertrophic area due to stem insertion, progression to a fracture during the postoperative period was possible. Therefore, we determined that using a sufficiently long stem to bridge the cortical hypertrophy and avoid postoperative stress concentration was essential.

THA in the presence of localized cortical hypertrophy and concomitant osteoarthritis of the same hip is rare, with few reported cases [5,18]. Yee et al. reported a case in a 72-year-old woman with a complete AFF in the subtrochanteric region associated with same-sided osteoarthritis, in which THA was performed using an uncemented stem and additional reinforcement with a plate and wiring [18]. Similarly, Joaquin et al. documented a 66-year-old woman in whom an incomplete AFF in the subtrochanteric region associated with osteoarthritis progressed to a complete AFF after THA, necessitating additional surgery with wiring fixation of the fracture site. The authors suggested bridging the fracture site with cemented or long revision stems combined with wire or lateral plate fixation [5]. However, these previous reports did not provide specific recommendations regarding the optimal stem length.

Strategies for managing Vancouver classification type B2 or B3 periprosthetic fractures around artificial hips may provide useful guidance for bridging the fracture site with a stem. The standard approach for type B2 or B3 fractures involves removing any existing stem loosened due to the fracture and replacing it with a sufficiently long stem that bridges the fracture site [19].

Several studies have proposed various options for stem selection. For example, using all porous-coated uncemented stems is recommended to bridge the fracture site with additional reinforcement using plates and wires or cemented stems [1,19]. Regarding stem length, biomechanical studies using model bones suggest that the revision stem should bypass the fracture site by at least two cortical diameters to ensure adequate stabilization [20,21,22].

The FEM simulations conducted in this study with various stem lengths yielded results consistent with those of previous reports, suggesting the use of sufficiently long stems to bridge vulnerable areas. The maximum load during daily activities must include the maximum stress during stair climbing. The transverse diameter of the cortical hypertrophy in the present case was 25 mm, and the bridging length of the cortical hypertrophy with a 150 mm long stem was 35 mm, corresponding to 1.4 times the femoral cortical diameter. This was 30% shorter than the two femoral cortex diameters recommended for fracture management in previous reports [20,21,22].

For localized cortical hypertrophy, as opposed to complete fractures, the axial stability of the implant bridging is less critical. Therefore, a shorter stem length should be adequate for bridging cortical hypertrophy, as indicated in this study. Given the limited variation in stem lengths available for clinical use, a stem > 150 mm in bridging length that is physically insertable should be used. The 175 mm stem used in this case was deemed to be of sufficient length from the perspective of the stress distribution.

Currently, the FEM is widely utilized in the medical field, particularly in orthopedics, playing a crucial role in understanding pathologies and designing patient-specific implants. However, effectively using the FEM requires expertise and technical skills. Additionally, access to licensed software and high-performance computational hardware is necessary, making it not readily available to everyone. Therefore, the cost-effectiveness of FEM should be carefully considered [23,24].

This study used data from a single female patient. For simplicity, we focused solely on the stem length, without analyzing other factors that could affect the stress distribution, such as stem shape, fixation concept, or diameter. The loading and boundary conditions for normal walking and stair climbing are based on simulations from previous studies, with a simplified representation of the soft tissue function. Ideally, verification through experimental studies with simulated or cadaveric bones would be beneficial; however, creating reproducible models for such validation is challenging. Thus, we recommend further validation using the FEM in multiple similar cases. Additionally, the postoperative observation period was limited to 3 months in the present case. Thus, as long-term stress distributions in cortical hypertrophy areas remain unknown, careful monitoring is necessary.

## 6. Conclusions

In the present case of femoral osteoarthritis with localized cortical hypertrophy in the subtrochanteric region, we performed FEM-based preoperative planning and a long stem revision for THA. From the perspective of the stress distribution, a stem length > 150 mm (a bridging length that is 1.4 times the femoral cortical diameter) is required to bridge the area of cortical hypertrophy. The 175 mm stem used in this case was sufficient for the distribution of stress.

## Figures and Tables

**Table 1:** Material parameters of each structure.

	Materials	Young’s Modulus (GPa)	Poisson’s Ratio
Femoral Bone	Heterogeneous model	Keyak (1998) [8]	0.40
Stem	Titanium alloy (Ti-6Al-4V)	109	0.28
Artificial head	Ceramic (Alumina)	350	0.23

**Table 2:** Loading during simulated “normal walking” and “stair-climbing” scenarios.

Normal Walking, Right, B.W (*N*) = 504						
Force	X (*N*)	Y (*N*)	Z (*N*)	Loading Point	(%)	Load (*N*)
Hip contact	−54.0	32.8	−229.2	P0	238	1199.52
ABD	−58.0	4.3	86.5	P1	104	
TFL-P	−7.2	11.6	13.2	P1	19	
TFL-D	0.5	−0.7	−19.0	P1	19	
P1 total force	64.7	−15.2	80.7	P1	105	529.2
VL	0.9	−18.5	−92.9	P2	95	478.8
Stair Climbing, Right, B.W (*N*) = 504						
Force	X (*N*)	Y (*N*)	Z (*N*)	Loading Point	(%)	Load (*N*)
Hip contact	−59.3	60.6	−236.3	P0	251	1265.04
ABD	−70.1	28.8	84.9	P1		
ITT-P	−10.5	3.0	12.8	P1		
ITT-D	0.5	−0.8	−16.8	P1		
TFL-P	−3.1	4.9	2.9	P1		
TFL-D	0.2	−0.3	−6.5	P1		
P1 total force	83.0	−35.6	77.3	P1	119	599.76
VL	2.2	−22.4	−135.1	P2	137	690.48
VM	8.8	39.6	−267.1	P3	270	1360.8

ABD: abductor; TFL: tensor fascia latae; -P: proximal; -D: distal; VL: vastus lateralis; VM: vastus medialis; ITT: iliotibialis tract; B.W: body weight.

**Table 3:** The number of components for each model.

Stem Length [mm]	Number of Elements
Control	411,681
90	589,149
100	595,689
110	618,600
120	631,146
130	640,215
140	656,649
150	667,911
160	673,071
170	681,366
180	681,891

## Data Availability

The data are available from the corresponding author upon reasonable request.

## References

[1] Shane E., Burr D., Abrahamsen B., Adler R.A., Brown T.D., Cheung A.M., Cosman F., Curtis J.R., Dell R., Dempster D.W. (2014). Atypical subtrochanteric and diaphyseal femoral fractures: Second report of a task force of the American Society for Bone and Mineral Research. J. Bone Min. Res..

[2] Min B.W., Koo K.H., Park Y.S., Oh C.W., Lim S.J., Kim J.W., Lee K.J., Lee Y.K. (2017). Scoring system for identifying impending complete fractures in incomplete atypical femoral fractures. J. Clin. Endocrinol. Metab..

[3] Cho J.W., Oh C.W., Leung F., Park K.C., Wong M.K., Kwek E., Kim H.J., Oh J.K. (2017). Healing of atypical subtrochanteric femur fractures after cephalomedullary nailing: Which factors predict union?. J. Orthop. Trauma..

[4] Giusti A., Hamdy N.A., Papapoulos S.E. (2010). Atypical fractures of the femur and bisphosphonate therapy: A systematic review of case/case series studies. Bone.

[5] Moya-Angeler J., Zambrana L., Westrich G.H., Lane J.M. (2016). Atypical femoral fracture post total hip replacement in a patient with hip osteoarthritis and an ipsilateral cortical thickening. Hip Int..

[6] Hirata Y., Inaba Y., Kobayashi N., Ike H., Fujimaki H., Saito T. (2013). Comparison of mechanical stress and change in bone mineral density between two types of femoral implant using finite element analysis. J. Arthroplast..

[7] Tano A., Oh Y., Fukushima K., Kurosa Y., Wakabayashi Y., Fujita K., Yoshii T., Okawa A. (2019). Potential bone fragility of mid-shaft atypical femoral fracture: Biomechanical analysis by a CT-based nonlinear finite element method. Injury.

[8] Keyak J.H., Rossi S.A., Jones K.A., Skinner H.B. (1998). Prediction of femoral fracture load using automated finite element modeling. J. Biomech..

[9] Bergmann G., Bender A., Dymke J., Duda G., Damm P. (2016). Standardized loads acting in hip implants. PLoS ONE.

[10] Heller M.O., Bergmann G., Kassi J.P., Claes L., Haas N.P., Duda G.N. (2005). Determination of muscle loading at the hip joint for use in pre-clinical testing. J. Biomech..

[11] Biemond J.E., Aquarius R., Verdonschot N., Buma P. (2011). Frictional and bone ingrowth properties of engineered surface topographies produced by electron beam technology. Arch. Orthop. Trauma. Surg..

[12] O’Shea K., Quinlan J.F., Kutty S., Mulcahy D., Brady O.H. (2005). The use of uncemented extensively porous-coated femoral components in the management of Vancouver B2 and B3 periprosthetic femoral fractures. J. Bone Jt. Surg. Br..

[13] Koh A., Guerado E., Giannoudis P.V. (2017). Atypical femoral fractures related to bisphosphonate treatment: Issues and controversies related to their surgical management. Bone Jt. J..

[14] Odvina C.V., Zerwekh J.E., Rao D.S., Maalouf N., Gottschalk F.A., Pak C.Y. (2005). Severely suppressed bone turnover: A potential complication of alendronate therapy. J. Clin. Endocrinol. Metab..

[15] Nishino T., Hyodo K., Matsumoto Y., Yanagisawa Y., Yamazaki M. (2024). Bisphosphonate- related atypical femoral fractures in patients with autoimmune disease treated with glucocorticoids: Surgical results for 20 limbs. J. Clin. Med..

[16] Black D.M., Geiger E.J., Eastell R., Vittinghoff E., Li B.H., Ryan D.S., Dell R.M., Adams A.L. (2020). Atypical femur fracture risk versus fragility fracture prevention with bisphosphonates. N. Engl. J. Med..

[17] Byun S.E., Lee K.J., Shin W.C., Moon N.H., Kim C.H. (2023). The effect of teriparatide on fracture healing after atypical femoral fracture: A systematic review and meta-analysis. Osteoporos. Int..

[18] Yee D., Cheng H.C. (2016). Hip arthroplasty for treatment of atypical femoral fracture with pre-existing hip osteoarthritis. J. Orthop. Trauma. Rehabil..

[19] Mondanelli N., Troiano E., Facchini A., Ghezzi R., Di Meglio M., Nuvoli N., Peri G., Aiuto P., Colasanti G.B., Giannotti S. (2022). Treatment algorithm of periprosthetic femoral fracturens. Geriatr. Orthop. Surg. Rehabil..

[20] Lewallen D.G., Berry D.J. (1998). Periprosthetic fracture of the femur after total hip arthroplasty: Treatment and results to date. Instr. Course Lect..

[21] Larson J.E., Chao E.Y., Fitzgerald R.H. (1991). Bypassing femoral cortical defects with cemented intramedullary stems. J. Orthop. Res..

[22] Panjabi M.M., Trumble T., Hult J.E., Southwick W.O. (1985). Effect of femoral stem length on stress raisers associated with revision hip arthroplasty. J. Orthop. Res..

[23] Quevedo González F.J., Nuño N. (2016). Finite element modelling approaches for well-ordered porous metallic materials for orthopaedic applications: Cost effectiveness and geometrical considerations. Comput. Methods Biomech. Biomed. Engin.

[24] Li J., Viceconti M., Li X., Bhattacharya P., Naimark D.M.J., Osseyran A. (2023). Cost-effectiveness analysis of CT-based finite element modeling for osteoporosis screening in secondary fracture prevention: An early health technology assessment in The Netherlands. MDM Policy Pract..

